# Accuracy and Outcomes of Computer-Aided Surgical Planning in Deep Circumflex Iliac Artery (DCIA) Free Flap Reconstruction of Maxillofacial Defects: A Systematic Review

**DOI:** 10.3390/jcm15124600

**Published:** 2026-06-13

**Authors:** Hyo-Joon Kim, Ji-Su Oh, Kun-Woo Kim, Jun-Seong Kim, Seong-Yong Moon

**Affiliations:** 1 Department of Oral and Maxillofacial Surgery, Chosun University Dental Hospital, Gwangju 61452, Republic of Korea; hyojoonkim@chosun.ac.kr (H.-J.K.); jsoh@chosun.ac.kr (J.-S.O.); 2 Dental Biomedical Engineering, College of Dentistry, Chosun University, Gwangju 61452, Republic of Korea; rjsdn0602@chosun.kr; 3 Clinical Coordinating Center, College of Dentistry, Chosun University, Gwangju 61452, Republic of Korea; jskim8190@chosun.ac.kr

**Keywords:** deep circumflex iliac artery, virtual surgical planning, computer-aided surgery, maxillofacial reconstruction, systematic review

## Abstract

**Background/Objectives**: Computer-aided surgical planning (CASP) technologies, including virtual surgical planning (VSP), 3D printed cutting guides, and patient-specific implants, have been increasingly applied to deep circumflex iliac artery (DCIA) free flap reconstruction of maxillofacial defects. Despite growing adoption, no systematic review has specifically evaluated their accuracy and clinical outcomes. This study aimed to comprehensively assess the impact of CASP on reconstruction accuracy, operative efficiency, flap survival, and implant rehabilitation in DCIA flap surgery. **Methods**: A systematic search of PubMed, Web of Science, and Google Scholar was conducted following Preferred Reporting Items for Systematic Reviews and Meta-Analyses (PRISMA) 2020 guidelines. Studies reporting CASP-assisted DCIA free flap reconstruction with three or more patients were included. Methodological quality was assessed using the Methodological Index for Non-Randomized Studies (MINORS) checklist and the Cochrane Risk of Bias 2.0 tool for the randomized controlled trial (RCT). **Results**: Thirty studies (1 RCT, 13 comparative, and 16 non-comparative) involving 844 patients were included. VSP with 3D-printed cutting guides was the most frequently used technology (n = 22). Mean linear deviations between planned and actual outcomes ranged from 0.40 to 4.4 mm, with most studies reporting 0.7–2.7 mm. The sole RCT demonstrated significantly better accuracy (1.3 vs. 5.5 mm, *p* < 0.001) and shorter reconstruction time (16 vs. 39 min, *p* < 0.001) with CASP. Flap survival ranged from 90% to 100%. **Conclusions**: CASP technologies, particularly VSP with 3D-printed cutting guides, appear to improve the accuracy and predictability of DCIA flap reconstruction. However, the evidence base is predominantly retrospective and heterogeneous; prospective multicenter studies with standardized outcome measures are needed before definitive clinical guidelines can be established.

## 1. Introduction

Maxillofacial reconstruction following tumor ablation, trauma, or congenital defects remains one of the most challenging procedures in oral and maxillofacial surgery [1]. The deep circumflex iliac artery (DCIA) free flap, first described by Taylor et al. in 1979 [2], has become an established option in maxillofacial reconstruction due to its favorable characteristics: adequate bone height and volume resembling the native mandible, potential for dental implant placement, and the ability to include a soft tissue component [3,4]. The iliac crest provides a bicortical bone stock that closely resembles the native mandibular cross-section, and the inclusion of the internal oblique muscle allows for simultaneous soft tissue reconstruction [3,5].

Despite these advantages, traditional DCIA flap surgery faces significant challenges. Intraoperative shaping of the iliac bone graft to match the complex three-dimensional (3D) geometry of maxillofacial defects is technically demanding, time-consuming, and prone to inaccuracies. Prolonged operative and ischemia times increase the risk of flap failure and postoperative complications [6]. Furthermore, the inability to precisely replicate the planned reconstruction often compromises functional outcomes, including occlusal rehabilitation with dental implants. Donor site morbidity, including gait disturbance and abdominal wall hernia, remains a concern with the DCIA flap [7].

Over the past two decades, computer-aided surgical planning (CASP) technologies have revolutionized reconstructive surgery. Virtual surgical planning (VSP) enables preoperative simulation of the osteotomy and reconstruction on 3D computed tomography (CT) models [8]. Patient-specific cutting guides, fabricated through 3D printing or selective laser sintering, transfer the virtual plan to the operative field with high fidelity [9]. Intraoperative navigation systems provide real-time guidance during bone shaping and positioning. More recently, patient-specific implants (PSI), including customized titanium reconstruction plates and meshes, have been developed using computer-aided design/computer-aided manufacturing (CAD/CAM) technology [10].

While the application of CASP to fibula free flap reconstruction has been extensively studied, including a published systematic review and meta-analysis by Barr et al. [11], no systematic review has specifically addressed the use of CASP technologies in DCIA flap reconstruction. The DCIA flap presents unique planning challenges distinct from those of the fibula: the curved anatomy of the iliac crest, the variable branching pattern of the DCIA pedicle, the proximity of the lateral femoral cutaneous nerve, and the need for precise 3D orientation to restore both bone continuity and adequate height for implant placement. These anatomical complexities make the DCIA flap a particularly compelling candidate for CASP integration.

The purpose of this systematic review is to comprehensively evaluate the current evidence on CASP technologies applied to DCIA free flap reconstruction of maxillofacial defects, with specific focus on: (1) accuracy of surgical execution compared to the virtual plan, (2) clinical outcomes including flap survival and complications, (3) operative efficiency (operation time, ischemia time), and (4) implant rehabilitation outcomes.

## 2. Materials and Methods

### 2.1. Protocol

This systematic review was conducted in accordance with the Preferred Reporting Items for Systematic Reviews and Meta-Analyses (PRISMA) 2020 guidelines [12]. The review protocol was not prospectively registered. The eligibility criteria, outcomes of interest, and the standardized data-extraction framework were defined a priori, before screening and data extraction commenced.

### 2.2. Eligibility Criteria

Inclusion criteria:

- Studies reporting DCIA or vascularized iliac crest free flap reconstruction for maxillofacial defects;
- Use of any form of computer-aided surgical planning (VSP, 3D printing, cutting guides, navigation, patient-specific implants, and CAD/CAM);
- Original research with ≥3 patients (to ensure minimum methodological rigor and reduce anecdotal bias);
- No language restriction.

Exclusion criteria:

- Case reports with fewer than three patients;
- Review articles, editorials, letters, and commentaries;
- Animal, cadaver, or phantom studies;
- Studies where DCIA-specific outcomes could not be separated from other flap types;
- Studies using DCIA without any computer-aided technology;
- Purely anatomical or imaging studies.

### 2.3. Information Sources and Search Strategy

A systematic search was conducted on 5 March 2026, using two electronic databases: PubMed/MEDLINE and Web of Science Core Collection. A supplementary search was performed using Google Scholar to ensure comprehensiveness. The search strategy combined terms related to the DCIA flap with terms related to computer-aided technologies. A supplementary search using Medical Subject Headings (MeSH) terms (“Surgery, Computer-Assisted” [MeSH], “Printing, Three-Dimensional” [MeSH], and “Computer-Aided Design” [MeSH]) was also performed in PubMed.

PubMed search string:

(“deep circumflex iliac artery” OR “DCIA flap” OR “iliac crest flap”

OR “iliac crest free flap” OR “vascularized iliac crest”)

AND

(“virtual surgical planning” OR “computer-aided” OR “computer-assisted”

OR “CAD” OR “CAD/CAM” OR “3D printing” OR “three-dimensional printing”

OR “patient-specific” OR “cutting guide” OR “surgical guide”

OR “navigation” OR “rapid prototyping” OR “stereolithograph*”)

The Web of Science search used equivalent terms adapted to its field syntax. Google Scholar was searched using simplified keyword combinations to capture any records missed by the primary databases. No date or language restrictions were applied. Reference lists of included studies were manually screened for additional relevant publications.

### 2.4. Study Selection

**Figure 1:**
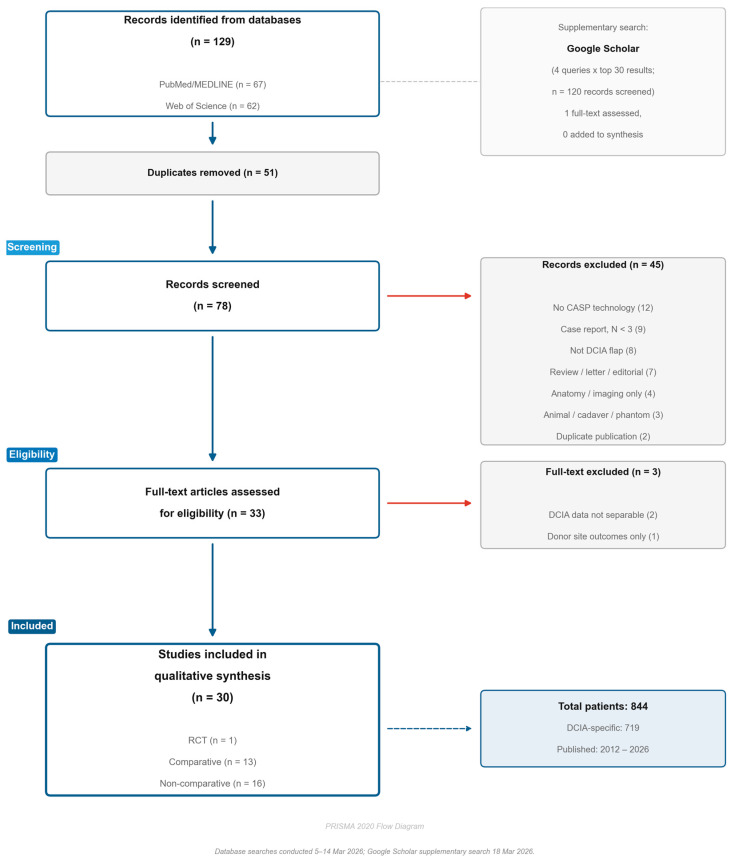
PRISMA flow diagram illustrating the study selection process.

Two reviewers (HJK and JSO) independently screened titles and abstracts for eligibility. Full-text articles were then assessed against the inclusion and exclusion criteria. Disagreements were resolved by consensus or consultation with the third author (SYM). The selection process is presented in the PRISMA flow diagram (Figure 1). For the two studies co-authored by members of the review team, eligibility decisions, data extraction, and quality assessment were performed independently by reviewers who were not authors of those studies (KWK and JSK) and were verified by a further non-conflicted reviewer; authors of the primary studies were excluded from extracting data or assessing the risk of bias for their own work.

### 2.5. Data Extraction

Data were extracted independently by two reviewers using a standardized form with 15 predefined fields: (1) study design, (2) sample size, (3) demographics, (4) diagnosis, (5) defect location, (6) CAD/CAM technology type, (7) software used, (8) accuracy metrics, (9) operation time, (10) ischemia time, (11) flap survival rate, (12) complications, (13) implant placement, (14) follow-up period, and (15) key findings.

### 2.6. Quality Assessment

Methodological quality was assessed using the Methodological Index for Non-Randomized Studies (MINORS) checklist [13]. Non-comparative studies were scored on 8 items (maximum 16 points), and comparative studies on 12 items (maximum 24 points). Each item was scored 0 (not reported), 1 (reported but inadequate), or 2 (reported and adequate). Studies scoring ≥12/16 (non-comparative) or ≥18/24 (comparative) were classified as “good” quality. The single randomized controlled trial was assessed using the Cochrane Risk of Bias 2.0 tool [14]. Quality assessment was performed independently by two reviewers (HJK and JSO), with disagreements resolved by consensus. Item-level (domain-level) MINORS scores for every included study are provided in Supplementary Table S1 so that the principal sources of bias, rather than total scores alone, can be appraised.

### 2.7. Data Synthesis

Due to substantial heterogeneity in study designs, sample sizes, CASP technologies used, and outcome measures reported, a meta-analysis was not feasible. Data were therefore synthesized narratively, organized by: (1) accuracy outcomes, (2) operative efficiency, (3) flap survival and complications, and (4) implant rehabilitation. Where the available data permitted, outcomes were additionally stratified by anatomical site (mandible versus maxilla) and by the type of CASP technology employed (including the use of intraoperative navigation). Given the narrow and heterogeneous evidence base, these comparisons are descriptive observations rather than formal subgroup or sensitivity analyses.

### 2.8. Use of Artificial Intelligence

AI-assisted tools (Claude Opus 4.7 (Anthropic, 2025) PBC, San Francisco, CA, USA, accessed 27 April 2026) were used for literature-screening support and English language editing. All content was reviewed, verified, and approved by all authors. AI was not used for data collection, analysis, or interpretation of results.

## 3. Results

### 3.1. Study Selection

The database search identified 129 records (PubMed: 67, Web of Science: 62). A supplementary Google Scholar search, comprising four simplified queries with the first approximately 30 records of each (approximately 120 records in total) screened, identified one additional potentially eligible study, which was assessed at full text but excluded because the use of CASP could not be confirmed; no additional studies were therefore added to the synthesis. After removing 51 duplicates, 78 unique records were screened by title and abstract. Forty-five records were excluded based on title/abstract screening (case reports with N < 3, reviews, letters, animal/cadaver studies, studies without DCIA or without CAD/VSP). The remaining 33 full-text articles were assessed for eligibility, of which three were excluded (two because DCIA-specific data were not separable from other flap types; one because the study primarily reported donor site outcomes rather than maxillofacial reconstruction accuracy). A total of 30 studies met the inclusion criteria and were included in the qualitative synthesis (Figure 1). The completed PRISMA 2020 checklist and the item-level methodological quality assessments are provided as Supplementary Materials.

### 3.2. Study Characteristics

Table 1 summarizes the characteristics of the 30 included studies [15,16,17,18,19,20,21,22,23,24,25,26,27,28,29,30,31,32,33,34,35,36,37,38,39,40,41,42,43,44]. The studies were published between 2012 and 2026, reflecting the relatively recent adoption of CASP technologies for DCIA reconstruction. One study was a randomized controlled trial (RCT) [15], 13 were comparative studies, and 16 were non-comparative case series. The total number of patients was 844, of whom 719 received DCIA flaps in the DCIA-specific study arms.

Study design: The majority were retrospective (24/30, 80.0%). Five studies employed a prospective non-comparative design, and one was a prospective RCT [15]. The 13 comparative studies were heterogeneous in their comparisons: six compared CASP-assisted versus conventional (freehand) techniques, four compared different CASP variants (e.g., multi-segmental vs. single-segmental design, complex vs. simple guides), two compared DCIA flaps with other flap types (fibula), and one employed a three-group design.

Geographic distribution: The majority of studies originated from China (n = 17, 56.7%), with the remainder from Europe (n = 8) and the Asia-Pacific region (n = 5).

Sample size: Individual study sample sizes ranged from three to 253 patients. The largest series (Lin et al., 2025) included 253 patients comparing multi-segmental and single-segmental DCIA flap designs [41].

Defect location: Mandibular reconstruction was performed in 24 studies, maxillary reconstruction in five studies, and mixed mandibular/maxillary reconstruction in one study.

### 3.3. Computer-Aided Technologies

The CASP technologies employed across the included studies are summarized in Table 2.

Virtual surgical planning (VSP): Used in 30/30 studies (1100.0%). VSP was performed on preoperative CT data to simulate osteotomy lines, bone segment positioning, and plate fixation.

3D-printed cutting guides were used in 27/30 studies (990.0%). Cutting guides were fabricated for both the recipient site (mandibular/maxillary osteotomy guides) and the donor site (iliac crest cutting guides).

3D-printed anatomical models were used in 17/30 studies (56.7%). Stereolithographic models served for prebending reconstruction plates and verifying the surgical plan.

Intraoperative navigation was used in 7/30 studies (23.3%). Navigation systems provided real-time guidance during bone positioning.

Patient-specific implants (PSI): Used in 3/30 studies (110.0%). PSI included customized titanium reconstruction plates fabricated by selective laser melting (SLM) and titanium meshes.

Software: Mimics (Materialise, Leuven, Belgium) was the most frequently used software for CT segmentation and planning (n = 16), followed by ProPlan CMF (Materialise) as the most common dedicated VSP platform (n = 14). iPlan CMF (BrainLab) was used in 5 studies, and Geomagic Control/Studio (3D Systems) was the predominant tool for postoperative accuracy analysis (n = 10).

### 3.4. Accuracy Outcomes

Quantitative accuracy data were reported in 17 of 30 studies (56.7%). Accuracy was measured using various methods, most commonly 3D surface-based analysis comparing preoperative virtual plans to postoperative CT scans using software such as Geomagic Control (3D Systems) or 3-matic (Materialise).

Linear deviation: Mean linear deviations ranged from 0.40 mm (Qiu et al. [19]) to 4.4 mm (Jie et al. [44]), with the majority of studies reporting values between 0.7 and 2.7 mm (Table 3). One outlier (Zhang M et al., 2019 [18]) reported osteotomy distances of 4.6–9.6 mm, which likely reflects a different measurement methodology rather than deviation from the surgical plan. Modabber et al. (2024) reported a surface-based mean deviation of 0.11 ± 0.04 mm for the DCIA flap shape using Geomagic Control [17]; however, the positional accuracy in the same study was considerably lower (height deviation 1.58 ± 1.88 mm, anteroposterior translation 5.38 ± 5.51 mm), illustrating that surface-shape comparison and positional accuracy represent distinct metrics. More broadly, the accuracy metrics reported across studies—surface-shape deviation, intercondylar distance, landmark-based linear deviation, osteotomy distance, and positional deviation—quantify fundamentally different constructs and cannot be interpreted on a common scale; the pooled range (0.40–4.4 mm) should therefore be read as a descriptive summary of heterogeneous measurements rather than as a single accuracy estimate. When the data were stratified by anatomical site, reported deviations in mandibular reconstruction (most studies 0.7–2.7 mm) and in the smaller maxillary subset (e.g., 0.40 mm) were broadly overlapping, although the limited maxillary data preclude firm site-specific conclusions. Among the seven studies that employed intraoperative navigation, reported accuracy spanned the entire observed range (0.40–4.4 mm), indicating that navigation use did not consistently correlate with improved accuracy and is therefore unlikely to be the dominant determinant of the reported outcomes.

Comparative accuracy: Among the comparative studies, two reported direct accuracy comparisons between CASP and conventional techniques, both demonstrating significantly better accuracy with CASP:

- Ayoub et al. (2014, RCT): intercondylar distance deviation 1.3 ± 0.2 mm vs. 5.5 ± 2.5 mm (*p* < 0.001) [15];
- Zheng et al. (2024): 1.45 ± 0.76 mm vs. 2.02 ± 0.89 mm (*p* = 0.034) [20].

Zhang et al. (2016) reported plan-to-outcome deviations of 0.70–1.92 mm for the CASP group and demonstrated significantly better clinical outcomes (mandibular contour, condyle position, dental restoration) compared to the conventional group (*p* < 0.05), although direct accuracy comparison was not possible as the conventional group did not have a virtual plan [21].

### 3.5. Operative Efficiency

Operation time: Reported in eight studies. Results were mixed; CASP did not consistently reduce total operation time but significantly reduced intraoperative reconstruction time. Ayoub et al. reported reconstruction time of 16.4 ± 6.7 min vs. 38.5 ± 10.0 min (*p* < 0.001) [15]. Zheng et al. (2024) reported a total operative time of 373 vs. 427 min (*p* = 0.001) [20].

Ischemia time: Reported in 2 DCIA-specific studies. CASP consistently reduced ischemia time:

- Ayoub et al. (2014): 96.1 ± 15.8 min vs. 122.9 ± 20.4 min (*p* < 0.005) [15];
- Modabber et al. (2012): reduction of 15.6 min with CASP [22].

### 3.6. Implant Rehabilitation

Implant placement data were reported in 18 studies. Among comparative studies, CASP-assisted groups demonstrated significantly higher rates of implant rehabilitation:

- Kang et al. (2021): 50% (VSP) vs. 11% (non-VSP) [23];
- Okcu et al. (2018): planned-to-inserted implant ratio 94% (CAD/CAM) vs. 50% (conventional) [24].

This finding may reflect the improved accuracy of bone positioning, which facilitates prosthetically driven implant placement. However, these comparative implant data are derived from only two studies, and confounding factors such as differences in follow-up duration, patient selection criteria for implant placement, and timing of implant surgery limit the strength of this conclusion.

### 3.7. Quality Assessment

The methodological quality of included studies is summarized in Table 4 and Table 5.

Non-comparative studies (n = 16): Mean MINORS score was 9.3/16 (range 6–12). One study scored ≥12/16 (good quality). Common weaknesses included lack of prospective data collection (12/16), absence of sample size calculation (16/16), and inadequate follow-up reporting (8/16).

Comparative studies (n = 13): Mean MINORS score was 16.6/24 (range 13–19). Five studies scored ≥18/24 (good quality). The main limitations were retrospective design (13/13), lack of sample size calculation (12/13), and sequential (non-contemporary) control groups (6/13).

RCT (n = 1): The Ayoub et al. study [15] was assessed as having “some concerns” overall on the Cochrane RoB 2.0 tool, primarily due to the inherent impossibility of blinding in surgical interventions. Randomization was performed using computer-generated allocation (RandList^®^), and outcomes were measured objectively using software-based 3D analysis.

## 4. Discussion

### 4.1. Principal Findings and Comparison with Existing Evidence

This systematic review is, to our knowledge, the first to comprehensively evaluate the role of CASP technologies specifically in DCIA free flap reconstruction of maxillofacial defects. Our analysis of 30 studies involving 844 patients suggests that CASP can improve reconstruction accuracy, reduce ischaemia time, and facilitate implant rehabilitation, although the strength of these inferences is limited by the predominantly retrospective and heterogeneous evidence base.

Across the included studies, the most consistent observation is a trend toward improved reconstruction accuracy with CASP, although the magnitude and measurement of this improvement vary substantially between studies. The sole RCT in this field, by Ayoub et al. [15], provides Level I evidence that computer-assisted DCIA reconstruction achieves significantly better accuracy (intercondylar distance deviation 1.3 vs. 5.5 mm) while reducing both ischemia time and reconstruction time. These findings are supported by the comparative study of Zheng et al. [20], which demonstrated a 28% reduction in linear deviation with CASP (1.45 vs. 2.02 mm), and by Zhang et al. [21], who reported significantly better clinical outcomes in the CASP group.

Compared to the fibula free flap literature, where Barr et al. [11] reported mean deviations of 1.0–2.0 mm with VSP-assisted fibula reconstruction, our findings suggest comparable benefits for DCIA flap reconstruction. However, the DCIA flap presents unique planning challenges, including the curved iliac crest anatomy, the variable position of the ascending branch of the DCIA, and the need to account for the internal oblique muscle component when chimeric flaps are harvested [3,17]. Unlike the fibula, which provides a linear bone stock amenable to straightforward segmental osteotomies, the iliac crest requires three-dimensional contouring to match the curvature of the mandible or the complex morphology of the maxilla. These challenges may explain the somewhat larger range of accuracy values (0.40–4.4 mm) compared to fibula studies, where deviations are generally reported below 2 mm.

The choice between DCIA and fibula free flaps remains surgeon- and case-dependent [45]. The DCIA flap may be preferred when adequate bone height is required for implant rehabilitation, when a bicortical bone stock is advantageous, or when soft tissue bulk from the internal oblique muscle is needed for intraoral lining [46,47]. CASP technologies may further expand the indications for DCIA flaps by mitigating their traditional disadvantages of complex donor site anatomy and technically demanding bone shaping.

### 4.2. Technology Evolution and Software Considerations

The included studies span from 2012 to 2026, reflecting a rapid evolution in CASP technology for DCIA flap surgery. Early studies primarily utilized 3D-printed anatomical models for plate prebending and surgical rehearsal. The introduction of patient-specific cutting guides marked a significant advance, enabling precise transfer of the virtual plan to the donor and recipient sites [17,23]. More recently, patient-specific titanium reconstruction plates fabricated by selective laser melting represent the latest evolution, eliminating the need for intraoperative plate bending entirely.

ProPlan CMF (Materialise) was the dominant planning software, used in nearly half of the included studies. This commercial platform provides an integrated workflow from CT segmentation through guide design to 3D printing. However, the cost associated with commercial VSP services has been cited as a potential barrier, particularly in resource-limited settings, although none of the included studies reported formal cost analyses. Several groups have explored surgeon-led or in-house planning protocols as lower-cost alternatives to commercial services [26,27,28], which may improve accessibility, although direct cost-effectiveness comparisons are lacking.

The postoperative accuracy analysis was most commonly performed using Geomagic Control (3D Systems), which provides surface-based deviation maps. However, the accuracy measurement methods varied considerably across studies: some reported intercondylar distance deviation, others used 3D surface superimposition, and several relied on landmark-based linear measurements. This heterogeneity is a major barrier to synthesizing evidence and underscores the need for consensus reporting guidelines. A standardized protocol specifying the timing of postoperative imaging (e.g., immediate vs. 6-month CT), the software and methodology for plan-to-outcome comparison, and a minimum set of accuracy metrics (e.g., mean 3D deviation, angular deviation, and volumetric difference) would greatly facilitate future meta-analyses.

An additional consideration is the learning curve associated with CASP adoption. Although no included study specifically evaluated this, institutions adopting CASP should anticipate a learning phase during which operative times may initially increase before the efficiency gains are realized.

### 4.3. Clinical Implications

Based on the current evidence, CASP technologies can be recommended for DCIA flap reconstruction, particularly when: (1) complex 3D defect geometry requires precise bone positioning; (2) implant-based dental rehabilitation is planned, as CASP groups demonstrated higher implant rehabilitation rates (50% vs. 11% patient-level implant rate [23]; 94% vs. 50% planned-to-inserted implant ratio [24]); (3) reduction in ischemia time is critical; and (4) training environments benefit from preoperative rehearsal.

The high flap survival rates (≥95% in most series) and the absence of reported CASP-attributable complications suggest that these technologies can be integrated into clinical practice, although, as noted above, the absence of reported complications should not be interpreted as evidence of an absence of risk. Notably, CASP may also influence donor site morbidity. By enabling more precise osteotomies and reducing the need for repeated bone trimming, CASP has the potential to minimize unnecessary bone removal from the iliac crest, preserve the anterior superior iliac spine, and reduce soft tissue dissection. Although donor site morbidity was not a primary outcome in most included studies, minimally invasive harvesting techniques facilitated by CASP, such as the medial approach described by Modabber et al. [22] and the myo-osseous chimeric technique by Bissinger et al. [28], represent promising developments.

The choice of specific CASP technology should be guided by institutional resources, surgeon experience, and defect complexity. For institutions, initiating CASP VSP with 3D-printed cutting guides represents a practical entry point that appears to improve accuracy without requiring navigation infrastructure. As experience grows, patient-specific implants and intraoperative navigation can be incorporated for more complex cases.

### 4.4. Limitations and Future Directions

Several limitations of the current evidence base should be acknowledged. The overwhelming majority of studies (80.0%) were retrospective, introducing selection and information bias. Most studies had small sample sizes (median N = 18.5), with six studies including only three or four patients, and no study performed a priori sample size calculation. Excluding these smallest studies (N < 5) would not alter the principal findings, as they contributed minimal patient numbers and their results were consistent with the overall trends. Substantial heterogeneity in CASP technologies, accuracy measurement methods, and outcome reporting precluded meta-analysis. Many studies reported short or unspecified follow-up periods, and the absence of negative studies suggests possible publication bias.

This review itself has limitations. The protocol was not prospectively registered in PROSPERO. Two databases (PubMed, Web of Science) supplemented by Google Scholar were searched; inclusion of additional databases such as Embase or Scopus may have identified further studies, although the supplementary Google Scholar search identified only one additional record, which was excluded after full-text assessment, suggesting adequate coverage. Although no language restriction was applied, the majority of included studies were published in English, with one Chinese-language study included [29]. Quality assessment was performed using MINORS, which has inherent limitations for surgical studies. Several further limitations warrant emphasis. First, a number of included studies originated from the same institutions or research groups—most notably the Chinese and German series—across adjacent time periods, raising the possibility of partially overlapping patient cohorts; where such overlap exists, the aggregate patient count may be overestimated, and the synthesis may be weighted toward a small number of high-volume centers. Second, CASP was applied as a heterogeneous bundle of technologies, and co-interventions such as intraoperative navigation varied across studies, so observed improvements in accuracy cannot be attributed to any single component. Third, the review combined mandibular and maxillary reconstructions, which differ substantially in geometry and reconstructive demands; although outcomes were stratified by anatomical site where data permitted, the small maxillary subset limits site-specific inference. Fourth, variation in surgeon experience and microsurgical skill—rarely reported in the included studies—may independently influence operative time, ischaemia time, and flap survival, further limiting the attribution of these outcomes to CASP. Finally, reported differences in implant rehabilitation rates may be confounded by patient selection, defect type, oncological status, radiotherapy, follow-up duration, and institutional protocols, and should not be interpreted as a direct effect of CASP alone.

Future research should prioritize: (1) multicenter prospective studies with standardized accuracy and outcome measures; (2) cost-effectiveness analyses comparing different CASP technologies; (3) long-term functional outcomes, including patient-reported outcome measures (PROMs); (4) development of consensus guidelines for CASP accuracy reporting; and (5) investigation of emerging technologies such as augmented reality navigation and robot-assisted osteotomy.

## 5. Conclusions

This systematic review suggests that computer-aided surgical planning technologies can improve the accuracy and predictability of DCIA free flap reconstruction for maxillofacial defects. The only RCT in this field confirms an approximately fourfold improvement in intercondylar distance accuracy, with reduced ischemia and reconstruction times. CASP facilitates implant-based dental rehabilitation by enabling prosthetically driven bone positioning and may reduce donor site morbidity through more precise osteotomies. On the basis of this predominantly retrospective and heterogeneous evidence, VSP with 3D-printed cutting guides appears to be a practical and accessible entry point for DCIA flap surgery, with patient-specific implants and navigation reserved for selected complex cases; these observations should be regarded as a cautious synthesis of promising but still limited evidence rather than as definitive clinical standards. However, the evidence base is predominantly retrospective with small sample sizes and heterogeneous outcome reporting. Future prospective multicenter studies with standardized accuracy metrics and long-term functional outcomes are needed to strengthen the evidence and guide clinical practice.

## Figures and Tables

**Table 1:** Characteristics of the 30 included studies.

Author (Year)	Country	Design	N (DCIA)	CASP/Control (n)	Indication	Site	Follow-Up	Study Period	Reconstruction Timing	Radiotherapy	Implant Rehab.
Zheng C (2026) [30]	China	Retro comp	31	31 (no control)	Mixed	Mandible	NR	2018–2023	NR	Excluded	Performed
Peters F (2024) [31]	Germany	Retro	20	20 (no control)	Mixed	Mandible	NR	2012–2021	immediate	Post (some)	Planned
Modabber A (2024) [17]	Germany	Retro comp	10	5/5	Mixed	Mandible	6 mo+	2015–2022	delayed	NR	Performed
Bissinger O (2025) [28]	Austria/Czech Republic	Prospective	8	8 (no control)	Mixed	Mandible	NR	NR	immediate	Not precluded	Performed
Dou CB (2025) [32]	China	Retro	10	10 (no control)	Mostly benign	Maxilla	NR	NR	immediate	NR	Performed
Zheng C (2024) [20]	China	Retro comp	44	23/21	Mixed	Mandible	NR	2015–2021	immediate	NR	Performed
Qiu SY (2023) [19]	China	Retro	11	11 (no control)	Mixed	Maxilla	NR	2018–2020	immediate	NR	Performed
Kang YF (2021) [23]	China	Retro comp	34	16/18	Mostly benign	Maxilla	NR	2017–2020	NR	Post (some)	Performed
Zhang WB (2016) [21]	China	Retro comp	45	15/30	Benign	Mandible	44 mo (6–98)	2008–2015	immediate	NR	Performed
Shen Y (2012) [33]	China	Retro	14	14 (no control)	Mixed	Mandible	18 mo (12–22)	2009–2010	mixed	Pre + post	Performed
Zheng L (2019) [34]	China	Retro	4	4 (no control)	Mixed	Mandible	NR	2015–2018	NR	NR	Planned
Li Y (2020) [35]	China	Retro	5	5 (no control)	Mixed	Mandible	10–28 mo	2016–2018	delayed	NR	Planned
Modabber A (2012) [22]	Germany	Retro comp	20	5/15	Mixed	Mandible	6 mo	NR	immediate	NR	Planned
Zho M (2019) [36]	China	Retro comp	27	14/13	Mixed	Mandible	NR	2013–2018	NR	NR	NR
Kim SR (2020) [37]	Republic of Korea	Retro comp	10	15 (no control)	Mixed	Mandible	12 mo+	2016–2018	NR	Post (some)	Planned
Thomas CV (2013) [38]	UK	Case series	4	4 (no control)	Malignant	Maxilla	NR	NR	NR	Post (some)	Planned
Wang LD (2023) [39]	China	Retro comp	8	17 (no control)	Mixed	Mandible	1 mo	2018–2022	NR	NR	NR
Ting JW (2014) [40]	Australia	Prospective	20	20 (no control)	Mostly malignant	Mandible	18 mo–4 yr	NR	NR	NR	NR
Lin H (2025) [41]	China	Retro comp	253	86/167	Mixed	Mandible	6 mo	2018–2022	immediate	NR	Performed
Wüster J (2025) [16]	Germany	Retro comp	43	56/56	Malignant	Mandible	4.0 yr (1.0–7.0)	2000–2019	mixed	NR	Planned
Yu Y (2020) [42]	China	Retro comp	20	20/10	Benign	Mandible	12–36 mo	2012–2019	NR	NR	Performed
Chen Y (2021) [29]	China	Prospective	4	4 (no control)	Mixed	Mandible	12–24 mo	2018–2019	immediate	NR	NR
Kim NK (2016) [25]	Republic of Korea	Retro	3	3 (no control)	Mixed	Mandible	1–5 mo	2013	immediate	NR	Planned
Ayoub N (2014) [15]	Germany	RCT	20	10/10	Mixed	Mandible	NR	NR	immediate	NR	Planned
Zhang M (2019) [18]	China	Retro	20	20 (no control)	Benign	Mandible	12–36 mo	2013–NR	Immediate	NR	Performed
Yao B (2019) [43]	China	Retro	4	4 (no control)	Trauma	Maxilla (bilat.)	6–42 mo	2014–2018	delayed	NR	Performed
Okcu Y (2018) [24]	Germany	Retro comp	10	10/10/10	Mixed	Mandible	NR	2012–2016	mixed	Postop (n = 4)	Performed
Jie B (2020) [44]	China	Retro	9	20 (no control)	Trauma	Mixed	24 mo (6–96)	2009–2019	delayed	NR	Performed
Kim HJ (2025) [26]	Republic of Korea	Prospective	5	5 (no control)	Mixed	Mandible	6 mo	2024–	immediate	Excluded	NR
Shin DH (2025) [27]	Republic of Korea	Prospective	3	4 (no control)	Benign	Mandible	6 mo	NR	NR	NR	NR

Study Design: RCT 1 (3.3%); Prospective non-comparative 5 (16.7%); Retrospective comparative 13 (43.3%); Retrospective non-comparative 10 (33.3%); Case series 1 (3.3%). Comparative study subtypes (n = 13): CASP vs. conventional 6; CASP variant vs. variant 4; DCIA vs. other flap 2; Three-group mixed 1. Geographic Distribution: China 17 (56.7%); Europe 8 (26.7%); Asia-Pacific 5 (16.7%). Total: 844 patients (DCIA-specific: 719). comp, comparative; mo, months; NR, not reported; Retro, retrospective; yr, years.

**Table 2:** Computer-aided surgical planning technologies.

Author (Year)	Planning Software	CASP Method	Cutting Guide	Navigation	Flap Config.
Zheng C (2026) [30]	NR	VSP + prebent plate	Yes	No	Myo-osseous
Peters F (2024) [31]	ProPlan CMF	VSP only	Yes	No	Osseous
Modabber A (2024) [17]	ProPlan CMF 3.01	VSP only	Yes	No	Osseous + TMJP
Bissinger O (2025) [28]	KLS Martin/TecuMed	VSP only	Yes	No	Myo-osseous chimeric
Dou CB (2025) [32]	NR	VSP only	Yes	No	Osseous/myo-osseous
Zheng C (2024) [20]	Mimics 19, Geomagic	VSP + prebent plate	Yes	No	Osseous
Qiu SY (2023) [19]	ProPlan CMF 3.0	VSP only	Yes	Yes	Osseous
Kang YF (2021) [23]	ProPlan CMF, iPlan	VSP + prebent plate	Yes	Yes	Osseous
Zhang WB (2016) [21]	Mimics, ProPlan, iPlan	VSP + prebent plate	Yes	Yes	Osseous/osteomusc.
Shen Y (2012) [33]	Mimics 8.1, Surgicase	VSP + prebent plate	Yes	No	Osseous
Zheng L (2019) [34]	ProPlan CMF 1.4	VSP + prebent plate	Yes	No	Osseous
Li Y (2020) [35]	Mimics 15, ProPlan 2.1	VSP + prebent plate	Yes	No	Osseous
Modabber A (2012) [22]	SurgiCase CMF, 3-matic	VSP + prebent plate	Yes	No	Osseous
Zho M (2019) [36]	Mimics 19, Geomagic	VSP + prebent plate	Yes	No	Osseous
Kim SR (2020) [37]	Aview, OnDemand3D	VSP only	Yes	No	Osseous
Thomas CV (2013) [38]	In-house	VSP only	Yes	No	Myo-osseous
Wang LD (2023) [39]	Mimics 19, ProPlan CMF	VSP + prebent plate	Yes	No	Osseous
Ting JW (2014) [40]	BrainLAB VectorVision	VSP + prebent plate	No	Yes	Mixed
Lin H (2025) [41]	Mimics 19.0	VSP + prebent plate	Yes	No	Oss./myo-oss./comp.
Wüster J (2025) [16]	NR	VSP + PSI	Yes	No	Osseous ± skin
Yu Y (2020) [42]	ProPlan, Mimics, iPlan	VSP + prebent plate	No	Yes	Osseous
Chen Y (2021) [29]	AccuNavi-A2.0	VSP only	Yes	No	DCIAPF chimeric
Kim NK (2016) [25]	Mimics, MeVisLab	VSP + prebent plate	Yes	No	Myo-osseous
Ayoub N (2014) [15]	ProPlan CMF, Geomagic	VSP + prebent plate	Yes	No	Osseous
Zhang M (2019) [18]	Mimics 8.0, Philips ISP	VSP + prebent plate	Yes	No	Osseous
Yao B (2019) [43]	ProPlan 3.0, Geomagic	VSP + prebent plate	No	Yes	Osseous
Okcu Y (2018) [24]	Xilloc Medical	VSP only	Yes	No	Osseous
Jie B (2020) [44]	ProPlan, SurgiCase CMF	VSP + prebent plate	Yes	Yes	Mixed
Kim HJ (2025) [26]	Mimics 18, 3-matic 13	VSP + PSI	Yes	No	Myo-osseous
Shin DH (2025) [27]	Mimics 18, Reconeasy	VSP + PSI	Yes	No	Myo-osseous

Technology Distribution: VSP + cutting guide 27 (90.0%); VSP + prebent plate 18 = 60.0%; Intraoperative navigation 7 (23.3%); Patient-specific implant/plate 3 (10.0%). Top Software: Mimics (Materialise) n = 16; ProPlan CMF (Materialise) n = 14; Geomagic (3D Systems) n = 8; 3-matic (Materialise) n = 5; iPlan/VectorVision (BrainLAB) n = 5. CAD/CAM, computer-aided design/manufacturing; DCIAPF, deep circumflex iliac artery perforator flap; NR, not reported; VSP, virtual surgical planning.

**Table 3:** Summary of accuracy outcomes.

Author (Year)	N	Measurement Method	CASP (mm)	Control (mm)	*p*
Ayoub N (2014) [15]	20	Intercondylar distance	1.3 ± 0.2	5.5 ± 2.5	<0.001
Qiu SY (2023) [19]	11	Surface deviation (Geomagic)	0.40 ± 0.08	—	—
Kim HJ (2025) [26]	5	Surface deviation (3-matic)	0.73 ± 0.23	—	—
Zhang WB (2016) [21]	45	Multiple (ICD, contour)	0.70–1.92	Worse (conv.)	<0.05
Shin DH (2025) [27]	3	Surface deviation (3-matic)	0.90 ± 0.34	—	—
Wang LD (2023) [39]	8	Color-coded map + landmarks	0.95–1.11	—	—
Modabber A (2024) [17]	10	3D surface shape (Geomagic) †	0.11 ± 0.04	—	—
Li Y (2020) [35]	5	3D deviation (Geomagic)	1.15 ± 1.68	—	—
Zheng C (2026) [30]	31	Superimposition + ICL/IGL	1.22–1.52	—	—
Peters F (2024) [31]	20	Osteotomy displacement (Geomagic)	1.3–2.7	—	—
Zheng C (2024) [20]	44	Digital overlap superimposition	1.45 ± 0.76	2.02 ± 0.89	0.034
Zho M (2019) [36]	27	Symmetry + gap + midline	1.6 ± 0.7 (CGT)	2.4 ± 1.2 (SGT)	0.02
Kim SR (2020) [37]	15	Landmark deviation	0.85–2.56	—	—
Yao B (2019) [43]	4	3D deviation (Geomagic)	2.67–4.32	—	—
Jie B (2020) [44]	20	3D deviation (Geomagic)	4.4 ± 0.8	—	—
Zheng L (2019) [34]	4	% within threshold	81–96% <3 mm	—	—
Zhang M (2019) [18] *	20	Osteotomy distance	4.6–9.6	—	—

* Zhang M 2019 values represent osteotomy distances, not deviation from virtual plan. † Modabber A 2024: 0.11 mm reflects surface-shape comparison only; positional accuracy was 1.58–5.38 mm. Studies reporting quantitative accuracy: 17/30 (56.7%). Range of mean deviations: 0.40–4.4 mm. Most studies: 0.7–2.7 mm. CASP, computer-aided surgical planning; CGT, complicated guiding template; ICD, intercondylar distance; ICL, intercondylar line; IGL, intergonial line; SGT, simple guiding template.

**Table 4:** Methodological quality assessment of non-comparative studies (MINORS).

Author (Year)	Score	Quality
Ting JW (2014) [40]	12	Good
Li Y (2020) [35]	11	Moderate
Dou CB (2025) [32]	10	Moderate
Qiu SY (2023) [19]	10	Moderate
Zheng L (2019) [34]	10	Moderate
Chen Y (2021) [29]	10	Moderate
Zhang M (2019) [18]	10	Moderate
Yao B (2019) [43]	10	Moderate
Jie B (2020) [44]	10	Moderate
Kim HJ (2025) [26]	10	Moderate
Peters F (2024) [31]	9	Moderate
Bissinger O (2025) [28]	9	Moderate
Shin DH (2025) [27]	9	Moderate
Shen Y (2012) [33]	8	Low
Thomas CV (2013) [38]	6	Low
Kim NK (2016) [25]	6	Low

Mean: 9.3/16 (range 6–12). Good (≥12): 1; Moderate (8–11): 12; Low (<8): 3.

**Table 5:** Methodological quality assessment of comparative studies (MINORS).

Author (Year)	Score	Quality
Zheng C (2026) [30]	19	Good
Zhang WB (2016) [21]	19	Good
Modabber A (2024) [17]	18	Good
Zheng C (2024) [20]	18	Good
Lin H (2025) [41]	18	Good
Kim SR (2020) [37]	17	Moderate
Zho M (2019) [36]	16	Moderate
Wüster J (2025) [16]	16	Moderate
Yu Y (2020) [42]	16	Moderate
Okcu Y (2018) [24]	16	Moderate
Kang YF (2021) [23]	15	Moderate
Wang LD (2023) [39]	15	Moderate
Modabber A (2012) [22]	13	Low

Mean: 16.6/24 (range 13–19). Good (≥18): 5; Moderate (14–17): 7; Low (<14): 1.

## Data Availability

No new data were created or analyzed in this study. Data sharing is not applicable to this article.

## Supplementary Materials

The following supporting information can be downloaded at: https://www.mdpi.com/article/10.3390/jcm15124600/s1, Table S1: Item-level (domain-level) MINORS quality assessment scores for all included studies; File S1: Completed PRISMA 2020 checklist.
