# Supplementary material for: Accuracy and Outcomes of Computer-Aided Surgical Planning in Deep Circumflex Iliac Artery (DCIA) Free Flap Reconstruction of Maxillofacial Defects: A Systematic Review
Source: J Clin Med. 2026 Jun 13;15(12):4600. doi: 10.3390/jcm15124600 (PMC13302490; doi:10.3390/jcm15124600)
Supplement: Supplementary file 1 [file jcm-15-04600-s001.zip › Supplementary_File_S1_PRISMA_2020_Checklist_v3.pdf]

PRISMA 2020 Checklist

Reference: Page MJ et al. The PRISMA 2020 statement: an updated guideline for reporting systematic reviews. BMJ 2021;372:n71. doi:10.1136/bmj.n71

Note: Section/page references correspond to the revised manuscript. "Amended" denotes content added in this revision (see Supplementary File - Manuscript Insertions). "Not applicable (NA)" entries indicate items not relevant to a narrative synthesis without meta-analysis; rationale is given inline. "NR" in tables denotes outcomes not reported by the original study.

Section / Topic	#	Checklist Item	Location in Manuscript
**TITLE**			
**Title**	1	Identify the report as a systematic review.	Title page - title explicitly contains "A Systematic Review".
**ABSTRACT**			
**Abstract**	2	See the PRISMA 2020 for Abstracts checklist (structured: Background/Objectives, Methods, Results, Conclusions).	Abstract - structured with Background/Objectives, Methods, Results, Conclusions.
**INTRODUCTION**			
**Rationale**	3	Describe the rationale for the review in the context of existing knowledge.	Section 1. Introduction, paragraphs 1-4 (DCIA flap background; conventional limitations; evolution of CASP; fibula precedent and DCIA-specific challenges).
**Objectives**	4	Provide an explicit statement of the objective(s) or question(s) the review addresses.	Section 1. Introduction, final paragraph - four explicit objectives: (1) accuracy of surgical execution compared to the virtual plan; (2) clinical outcomes including flap survival and complications; (3) operative efficiency (operation time, ischemia time); and (4) implant rehabilitation outcomes. Characterization of CASP technologies and software is reported in Results Section 3.3.
**METHODS**			
**Eligibility criteria**	5	Specify the inclusion and exclusion criteria for the review and how studies were grouped for the syntheses.	Section 2.2 Eligibility Criteria - itemized inclusion (4 criteria) and exclusion (6 criteria) lists.
**Information sources**	6	Specify all databases, registers, websites, organisations, reference lists and other sources searched or consulted to identify studies. Specify the date when each source was last searched or consulted.	Section 2.3 - PubMed/MEDLINE, Web of Science Core Collection, and Google Scholar (supplementary). Last searched 5 March 2026. Manual reference-list screening of included studies.
**Search strategy**	7	Present the full search strategies for all databases, registers and websites, including any filters and limits used.	Section 2.3 - full PubMed search string presented verbatim; Web of Science equivalent terms described; Google Scholar simplified keyword combinations; no date or language restrictions.
**Selection process**	8	Specify the methods used to decide whether a study met the inclusion criteria of the review, including how many reviewers screened each record and each report retrieved, whether they worked independently, and if applicable, details of automation tools used in the process.	Section 2.4 Study Selection - two reviewers (HJK, JSO) independently screened titles/abstracts and full texts; disagreements resolved by consensus or third author (SYM). Section 2.8 - AI tools (Claude, Anthropic) used only as a screening support; all decisions verified by authors.

**Data collection process**	9	Specify the methods used to collect data from reports, including how many reviewers collected data from each report, whether they worked independently, any processes for obtaining or confirming data from study investigators, and if applicable, details of automation tools used in the process.	Section 2.5 Data Extraction - two reviewers extracted independently using a standardized 15-field form; no contact with original investigators was required.
**Data items**	10a	List and define all outcomes for which data were sought. Specify whether all results that were compatible with each outcome domain in each study were sought (e.g. for all measures, time points, analyses), and if not, the methods used to decide which results to collect.	Section 2.5 + Section 2.7 - outcomes grouped for synthesis: (1) accuracy (linear/angular deviation); (2) operative efficiency (operation time, ischemia time); (3) flap survival and complications; (4) implant rehabilitation rate. All compatible measures within each domain were extracted.
**Data items**	10b	List and define all other variables for which data were sought (e.g. participant and intervention characteristics, funding sources). Describe any assumptions made about any missing or unclear information.	Section 2.5 - 15 predefined fields: (1) study design, (2) sample size, (3) demographics, (4) diagnosis, (5) defect location, (6) CAD/CAM technology type, (7) software used, (8) accuracy metrics, (9) operation time, (10) ischemia time, (11) flap survival rate, (12) complications, (13) implant placement, (14) follow-up period, and (15) key findings. Missing items reported as "NR" in tables.
**Study risk of bias assessment**	11	Specify the methods used to assess risk of bias in the included studies, including details of the tool(s) used, how many reviewers assessed each study and whether they worked independently, and if applicable, details of automation tools used in the process.	Section 2.6 Quality Assessment (amended) - two reviewers independently assessed methodological quality with disagreements resolved by consensus. MINORS checklist for non-randomized studies (8 items, max 16 for non-comparative; 12 items, max 24 for comparative); Cochrane RoB 2.0 for the single RCT. No automation tools were used for risk-of-bias assessment.
**Effect measures**	12	Specify for each outcome the effect measure(s) (e.g. risk ratio, mean difference) used in the synthesis or presentation of results.	Section 2.7 (amended) - continuous outcomes (accuracy, operative time, ischemia time) summarized as mean +/- SD with study-reported P values for between-group comparisons; categorical outcomes (flap survival, implant rehabilitation) summarized as proportions.
**Synthesis methods**	13a	Describe the processes used to decide which studies were eligible for each synthesis.	Section 2.7 - all eligible studies grouped by outcome domain (accuracy, efficiency, survival, implants); comparative studies separated for direct CASP vs. conventional contrast.
**Synthesis methods**	13b	Describe any methods required to prepare the data for presentation or synthesis, such as handling of missing summary statistics, or data conversions.	Section 2.7 - no quantitative data conversions performed; missing summary statistics flagged "NR" in tables and excluded from numeric ranges.
**Synthesis methods**	13c	Describe any methods used to tabulate or visually display results of individual studies and syntheses.	Section 3 - Tables 1 (study characteristics), 2 (CASP technologies), 3 (accuracy outcomes), 4 (MINORS non-comparative), 5 (MINORS comparative); Figure 1 (PRISMA flow diagram).
**Synthesis methods**	13d	Describe any methods used to synthesize results and provide a rationale for the choice(s).	Section 2.7 - narrative synthesis only. Rationale: substantial heterogeneity in study designs, sample sizes, CASP technologies, and outcome measures precluded meta-analysis.
**Synthesis methods**	13e	Describe any methods used to explore possible causes of heterogeneity among study results (e.g. subgroup analysis, meta-regression).	Not applicable (no meta-analysis). Sources of heterogeneity discussed narratively in Section 4 (Technology Evolution and Software Considerations).
**Synthesis methods**	13f	Describe any sensitivity analyses conducted	Not applicable (no meta-analysis). Outlier

		to assess robustness of the synthesized results.	accuracy values discussed individually in Section 3.4 (Zhang M 2019; Modabber 2024).
**Reporting bias assessment**	14	Describe any methods used to assess risk of bias due to missing results in a synthesis (arising from reporting biases).	Not formally assessed (precluded by absence of meta-analysis); selective outcome reporting discussed qualitatively in Section 4 Limitations.
**Certainty assessment**	15	Describe any methods used to assess certainty (or confidence) in the body of evidence for an outcome.	Formal GRADE assessment was not performed; methodological quality at the study level was assessed using MINORS and Cochrane RoB 2.0 (Section 2.6) and overall certainty discussed narratively in Section 4 Limitations.
**RESULTS**			
**Study selection**	16a	Describe the results of the search and selection process, from the number of records identified in the search to the number of studies included in the review, ideally using a flow diagram.	Section 3.1 - 129 records identified (PubMed 67, Web of Science 62); 51 duplicates removed; 78 screened; 45 excluded at title/abstract; 33 full-text assessed; 3 further excluded; 30 included. Figure 1 PRISMA flow diagram.
**Study selection**	16b	Cite studies that might appear to meet the inclusion criteria, but which were excluded, and explain why they were excluded.	Section 3.1 - 3 studies excluded at full-text review: 2 because DCIA-specific outcomes could not be separated from other flap types; 1 because the study primarily reported donor-site outcomes rather than maxillofacial reconstruction accuracy. Numerical exclusion reasons summarized in PRISMA flow diagram (Figure 1). Full citation list of records excluded at the full-text eligibility stage is deposited at the OSF registration (osf.io/[ID-to-be-inserted]).
**Study characteristics**	17	Cite each included study and present its characteristics.	Section 3.2; Table 1 - characteristics of all 30 included studies (references 15-44).
**Risk of bias in studies**	18	Present assessments of risk of bias for each included study.	Section 3.7; Table 4 (MINORS non-comparative, n=16); Table 5 (MINORS comparative, n=13); RCT RoB 2 assessment in Section 3.7.
**Results of individual studies**	19	For all outcomes, present, for each study: (a) summary statistics for each group (where appropriate) and (b) an effect estimate and its precision (e.g. confidence/credible interval), ideally using structured tables or plots.	Sections 3.4-3.6 with Table 3 (accuracy outcomes per study) and narrative reporting of operation time, ischemia time, flap survival, and implant rehabilitation per included study where reported.
**Results of syntheses**	20a	For each synthesis, briefly summarise the characteristics and risk of bias among contributing studies.	Sections 3.4-3.6 - characteristics and limitations of contributing studies summarized per outcome domain.
**Results of syntheses**	20b	Present results of all statistical syntheses conducted.	No meta-analysis. Narrative synthesis with reported ranges and direction of effect (e.g., CASP vs. conventional accuracy in Section 3.4; ischemia time reduction in Section 3.5).
**Results of syntheses**	20c	Present results of all investigations of possible causes of heterogeneity among study results.	Not applicable. Section 4 discusses sources of heterogeneity (technology generation, software platform, measurement method).
**Results of syntheses**	20d	Present results of all sensitivity analyses conducted to assess the robustness of the synthesized results.	Not applicable. Outliers handled by narrative discussion (Section 3.4 - Zhang M 2019; Modabber 2024).
**Reporting biases**	21	Present assessments of risk of bias due to missing results (arising from reporting biases) for each synthesis assessed.	Discussed narratively in Section 4 Limitations.
**Certainty of evidence**	22	Present assessments of certainty (or confidence) in the body of evidence for each outcome assessed.	Section 3.7 (MINORS distribution: non-comparative mean 9.3/16; comparative mean 16.6/24); overall body-of-evidence certainty discussed narratively in Section 4 Limitations. Formal GRADE assessment not undertaken (see item 15).

DISCUSSION			
**Discussion**	23a	Provide a general interpretation of the results in the context of other evidence.	Section 4 Principal Findings and Comparison with Existing Evidence (including comparison with Barr et al. fibula systematic review).
**Discussion**	23b	Discuss any limitations of the evidence included in the review.	Section 4 Limitations and Future Directions, paragraph 1 - retrospective design dominance, small samples, lack of standardized accuracy reporting.
**Discussion**	23c	Discuss any limitations of the review processes used.	Section 4 Limitations and Future Directions, paragraph 2 - protocol not prospectively registered (retrospectively deposited at OSF); two primary databases plus Google Scholar; potential publication bias.
**Discussion**	23d	Discuss implications of the results for practice, policy, and future research.	Section 4 Clinical Implications; Section 4 Future Directions; Section 5 Conclusions.
**OTHER INFORMATION**			
**Registration and protocol**	24a	Provide registration information for the review, including the register name and registration number, or state that the review was not registered.	Section 2.1 Protocol (amended) - review retrospectively registered at the Open Science Framework (OSF): osf.io/[ID-to-be-inserted]. Initial submission noted protocol was not prospectively registered (Section 4 Limitations).
**Registration and protocol**	24b	Indicate where the review protocol can be accessed, or state that a protocol was not prepared.	Section 2.1 (amended) - protocol deposited retrospectively at OSF (osf.io/[ID-to-be-inserted]). A prospective written protocol was not separately prepared or published.
**Registration and protocol**	24c	Describe and explain any amendments to information provided at registration or in the protocol.	Not applicable - review conducted as initially planned; retrospective registration reflects the executed methodology without amendment.
**Support**	25	Describe sources of financial or non-financial support for the review, and the role of the funders or sponsors in the review.	Funding statement (amended) - this study was supported by a research fund from Chosun University Dental Hospital (2024). The funder had no role in the design of the review, in the collection, analysis or interpretation of data, or in the preparation of the manuscript.
**Competing interests**	26	Declare any competing interests of review authors.	Conflicts of Interest statement - S.-Y.M. discloses inclusion of own published studies (Kim 2025, Shin 2025). Other authors declare no conflicts.
**Availability of data, code and other materials**	27	Report which of the following are publicly available and where they can be found: template data collection forms; data extracted from included studies; data used for all analyses; analytic code; any other materials used in the review.	Data Availability Statement (amended) - the review protocol, the 15-field data extraction form template, the data extracted from the included studies underlying Tables 1-5, and the methodological quality scoring sheets (MINORS, RoB 2) are deposited at the Open Science Framework (osf.io/[ID-to-be-inserted]) and are additionally available from the corresponding author on reasonable request. No new primary patient-level data were generated by this review.
