# Supplementary material for: Accuracy and Outcomes of Computer-Aided Surgical Planning in Deep Circumflex Iliac Artery (DCIA) Free Flap Reconstruction of Maxillofacial Defects: A Systematic Review
Source: J Clin Med. 2026 Jun 13;15(12):4600. doi: 10.3390/jcm15124600 (PMC13302490; doi:10.3390/jcm15124600)
Supplement: Supplementary file 1 [file jcm-15-04600-s001.zip › Supplementary_Table_S1_MINORS_itemlevel.pdf]

Supplementary Table S1. Item-level methodological quality assessment

Domain/item-level MINORS scores for non-randomized studies and Cochrane RoB 2.0 domain judgements for the single randomized trial. Per-item totals reconcile to the summary scores reported in the main manuscript (Section 3.4) and to the previously submitted Supplemental Digital Content. Scoring per MINORS: 0 = not reported; 1 = reported but inadequate; 2 = reported and adequate.

A. Non-comparative studies (MINORS items 1–8, maximum 16)

Items: (1) Clearly stated aim · (2) Inclusion of consecutive patients · (3) Prospective collection of data · (4) Endpoints appropriate to the study aim · (5) Unbiased assessment of the study endpoint · (6) Follow-up period appropriate · (7) Loss to follow-up < 5% · (8) Prospective calculation of the sample size.

Study (Year)	1	2	3	4	5	6	7	8	Total /16
**Ting JW (2014)**	2	2	2	2	1	2	1	0	12
**Li Y (2020)**	2	1	0	2	2	2	2	0	11
**Dou CB (2025)**	2	2	0	2	1	2	1	0	10
**Qiu SY (2023)**	2	1	0	2	2	1	2	0	10
**Zheng L (2019)**	2	1	0	2	2	1	2	0	10
**Chen Y (2021)**	2	1	2	1	0	2	2	0	10
**Zhang M (2019)**	2	1	0	2	1	2	2	0	10
**Yao B (2019)**	2	1	0	2	2	1	2	0	10
**Jie B (2020)**	2	1	0	2	2	1	2	0	10
**Kim HJ (2025)**	2	1	2	2	1	1	1	0	10
**Peters F (2024)**	2	2	0	2	1	1	1	0	9
**Bissinger O (2025)**	2	1	2	1	1	0	2	0	9
**Shin DH (2025)**	2	1	2	1	1	1	1	0	9
**Shen Y (2012)**	2	1	0	1	0	2	2	0	8
**Thomas CV (2013)**	2	1	0	1	0	0	2	0	6
**Kim NK (2016)**	2	1	0	1	0	0	2	0	6

B. Comparative studies (MINORS items 1–12, maximum 24)

Items 1–8 as above; (9) Adequate control group · (10) Contemporary groups · (11) Baseline equivalence of groups · (12) Adequate statistical analysis.

Study (Year)	1	2	3	4	5	6	7	8	9	10	11	12	T /24
**Zheng C (2026)**	2	2	0	2	2	2	2	0	1	2	2	2	19
**Zhang WB (2016)**	2	2	0	2	2	2	2	0	2	1	2	2	19
**Modabber A (2024)**	2	1	0	2	2	2	2	0	2	2	1	2	18
**Zheng C (2024)**	2	2	0	2	2	1	2	0	2	1	2	2	18
**Lin H (2025)**	2	2	0	2	2	1	1	0	2	2	2	2	18
**Kim SR (2020)**	2	1	0	2	2	2	1	0	2	2	1	2	17
**Zho M (2019)**	2	1	0	2	2	0	2	0	2	2	1	2	16
**Wüster J (2025)**	2	2	0	2	1	2	1	0	2	1	1	2	16
**Yu Y (2020)**	2	1	0	2	1	0	2	0	2	2	2	2	16
**Okcu Y (2018)**	2	1	0	2	1	2	1	0	2	2	1	2	16
**Kang YF (2021)**	2	1	0	2	2	0	1	0	2	1	2	2	15
**Wang LD (2023)**	2	1	0	2	2	1	2	0	2	2	0	1	15
**Modabber A (2012)**	2	1	0	1	1	1	2	0	2	1	1	1	13

C. Randomized controlled trial — Cochrane Risk of Bias 2.0 (Ayoub 2014)

Study	D1 Randomization	D2 Deviations from intended	D3 Missing outcome data	D4 Measurement	D5 Selection of the reported	Overall

	process	interventions		of the outcome	result	
**Ayoub N (2014)**	Some concerns	Some concerns	Low	Some concerns	Low	Some concerns

Domain-level rationale (RoB 2.0). D1 — randomisation by RandList® stated, but allocation concealment not described → Some concerns. D2 — open-label surgical intervention; objective outcome measurement mitigates risk → Some concerns. D3 — one flap failure per group, all participants accounted for → Low. D4 — outcomes measured by Geomagic software (objective), but outcome assessors not reported as blinded → Some concerns. D5 — pre-specified endpoints (trial registered as DRKS00005181) → Low. Overall judgement: Some concerns, driven by absence of explicit blinding and allocation-concealment reporting that are inherent to a surgical RCT.

Notes

• Item 3 (prospective collection): scored 0 for all retrospective designs and 2 for studies explicitly described as prospective.

• Item 5 (unbiased endpoint assessment): scored 2 where objective software-based measurement (e.g., Geomagic, 3-matic) was used as the primary endpoint per the MINORS authors' convention; 1 where assessment was clinical or single-observer; 0 where no objective measurement was reported.

• Item 8 (prospective sample-size calculation): scored 0 for all studies, as none reported an a priori calculation.

• Summary statistics derived from this table: non-comparative mean 9.3/16 (range 6–12, n = 16); comparative mean 16.6/24 (range 13–19, n = 13); RCT overall RoB Some concerns. These match the values reported in Section 3.4 of the main manuscript and in the previously submitted Supplemental Digital Content.
